# Cerebral neurocysticercosis mimicking or comorbid with episodic migraine?

**DOI:** 10.1186/1471-2377-14-138

**Published:** 2014-07-01

**Authors:** Yannick Fogoum Fogang, Massaman Camara, Amadou Gallo Diop, Mansour Mouhamadou Ndiaye

**Affiliations:** 1 Neurology Department, Fann Teaching Hospital, Cheikh Anta Diop University, Dakar, Senegal

**Keywords:** Neurocysticercosis, Migraine, Headache, Neuroinflammation

## Abstract

**Background:**

Neurocysticercosis is a major cause of neurological symptoms in developing countries. We report a case of cerebral neurocysticercosis presenting as episodic migraine without aura, with clinico-radiological correlations and discuss the possible causal influence of neurocysticercosis on the pathomechanisms of migraine.

**Case presentation:**

We report a 24 year-old male consulting for a one year history of recurrent headaches. He described bilateral frontal and/or temporal attacks of throbbing headache, moderate to severe in intensity, worsened by head movements and accompanied by nausea, photophobia and phonophobia. Attacks lasted between 12 and 60 hours if untreated. He never had symptoms suggestive of a migraine aura or an epileptic seizure. Headache attacks progressively increased in frequency to up to 5 to 7 severe attacks per month. On taking history, the patient reported having consumed undercooked porcine meat. Physical examination was unremarkable. A brain CT scan showed two contiguous occipital cystic lesions with ring enhancement and surrounding edema suggestive of cerebral neurocysticercosis. On laboratory work-up, blood serology for cysticercal antibodies was positive. Full blood count, erythrocyte sedimentation rate, c - reactive protein level, human immunodeficiency virus serology, liver and hepatic function were all normal. Albendazole (1000 mg/day) and prednisolone (60 mg/day) were prescribed for seven days. The patient was examined again two and six months after the end of his treatment and there was a significant reduction in headache severity and frequency.

**Conclusion:**

We propose that in our patient the occipital neurocysticercosis lesions cause migraine without aura-like attacks via inflammation in the surrounding brain parenchyma leading to sensitization of the trigemino-vascular system. We cannot rule out, however, the possibility that our patient has a genetic predisposition for migraine without aura and that the fortuitous association of neurocysticercosis is simply an aggravating factor of his migraine.

## Background

Neurocysticercosis is a major cause of neurological symptoms in developing countries such as seizures, focal deficits, cognitive impairment or headache [1]. Headaches in neurocysticercosis classically occur in the setting of increased intracranial pressure in patients with multiple parenchymal brain cystic lesions, or of hydrocephalus or giant subarachnoid cysts [2].

Primary headaches (migraine and tension-type headache) seem to be more prevalent in patients with neurocysticercosis [3,4]. It is not known, however, if there is a pathophysiological link between the 2 conditions or they are just comorbid because of their high prevalence.

We report a case of cerebral neurocysticercosis presenting as episodic migraine without aura, with clinico-radiological correlations and discuss the possible causal influence of neurocysticercosis on the pathomechanisms of migraine.

## Case presentation

A 24 year-old right handed male consulted the neurology outpatient clinic for recurrent headaches evolving since one year. He described bilateral frontal and/or temporal attacks of throbbing headache, moderate to severe in intensity, worsened by head movements and accompanied by nausea, photophobia and phonophobia. The headaches were alleviated by rest and paracetamol 1 g tid he took no more than 10 days per month. Attacks lasted between 12 and 60 hours if untreated. There was no identified trigger factor. He never had symptoms suggestive of a migraine aura or an epileptic seizure. He reported a mean frequency of 2 to 3 attacks per month during the first 5 months of appearance of the headaches whereafter attacks progressively increased in frequency up to 5 to 7 severe attacks per month, which significantly impacted on his social and professional life. On taking history, the patient reported having consumed undercooked porcine meat. He had no history of head trauma, weight loss or chronic medical condition. He had no previous personal or family history of headaches or migraine.

General physical examination was normal. Blood pressure was 115/70, heart rate 68 beats per minute, respiratory frequency 15 cycles per minute and axillary temperature of 36.8°C. The patient weight was 71 Kg.

**Figure 1:**
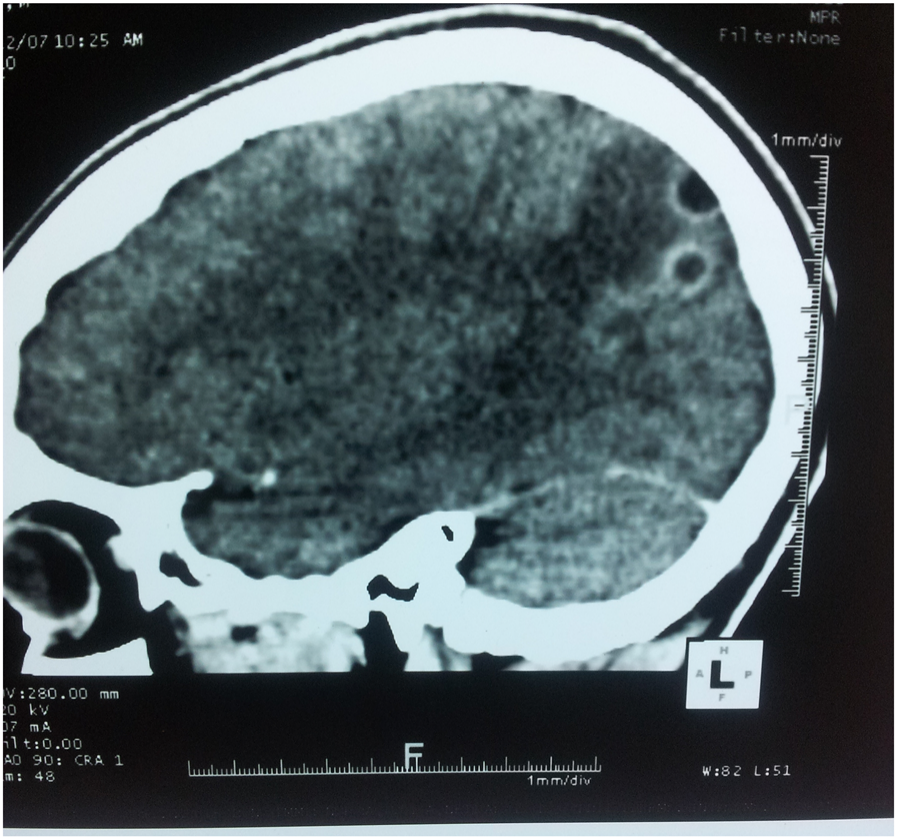
Contrast-enhanced CT showing two occipital cystic images with ring enhancement and perilesional edema.

Neurological examination was totally normal. Cranial nerve examination was normal; there were no motor, sensory or cerebello-vestibular abnormalities. There was no neck stiffness or Kernig’s sign. Temporo-mandibular joint examination and sinus point palpation were normal. There was no neck or pericranial tenderness on palpation.The EEG was unremarkable. Neuroimaging was scheduled to rule out a secondary headache. Brain CT scan showed two contiguous occipital cystic lesions with ring enhancement and surrounding edema suggestive of cerebral neurocysticercosis (Figure 1). On laboratory work-up, blood serology for cysticercal antibodies was positive. Full blood count, erythrocyte sedimentation rate, c-reactive protein level, human immunodeficiency virus serology, liver and hepatic function were all normal.

Albendazole (1000 mg/day) and prednisolone (60 mg/day) were prescribed for seven days. He was asked to take ibuprofen 600 mg bid during headache attacks. A migraine calendar was provided to the patient to monitor the headaches.

The patient was examined again two months after the end of his treatment. There was a reduction in headache frequency (from 5–7 to 2 attacks/month) and severity. A control brain CT scan was proposed, but could not be afforded by the patient. Six months after treatment of neurocysticercosis, the patient is still presenting 1 to 2 attacks/month, with no interference with his daily activities and easily relieved by ibuprofen intake.

## Discussion

Migraine is a complex and heterogeneous disorder, in which genetics and environment interact to generate dysfunctions at several levels of the central nervous system. It is the most frequent neurologic disorder worldwide [5]. Migraine has a genetic polymorphism which determines a dynamic threshold that can be modified by non genetic factors (stress, sleep deprivation, hormones, hypoglycemia etc.) [6]. However, there is no known pathophysiological rationale to explain the development of reccurent headaches as the result of parenchymal brain calcifications or a single granuloma. As the pathophysiology of migraine is also far to be well understood, patients with cerebral neurocysticercosis developing recurrent migrainous headaches could represent a novel study model to help unravel this issue. The conjunction of a history of regular consumption of undercooked porcine meat, suggestive features on CT scan, and presence of serum antibodies for cysticercosis leaves no doubt about the diagnosis of neurocysticercosis [2].

The headaches reported by this patient fulfill all the diagnostic criteria for migraine without aura (ICHD-3 beta 1.1.) [7] except for criterion E. The latter requires indeed that the headache can “Not better accounted for by another ICHD-3 diagnosis”. The question arises therefore whether the recurring headaches in this patient can be attributed to the neurocysticercosis in which case the diagnostic code would be “9.1.3 Headache attributed to intracranial fungal or other parasitic infection” or whether both conditions are simply associated by chance because of their high prevalence in the population [2,5]. The fact that the headaches probably developed and worsened in temporal relationship with neurocysticercosis and alleviated after specific treatment argues in favour of neurocysticercosis-induced migraine (ICHD-3 9.1.3). Several population- [3,4] and clinic-based [8-10] studies have shown that primary headaches are more prevalent in subjects with neurocysticercosis. Neurocysticercosis with occipital lesions can even mimick migraine with visual aura [11-13], which was not the case in our patient.

Unfortunately, we were not able to control the brain CT scan in this patient after anti-parasitic treatment neither to follow-up the patient for a sufficient time to verify eventual disapperance of his headache attacks. We can therefore not make any definitive statement about the final diagnosis.

Migraine is a disorder where a genetic predisposition sets a threshold for attacks that is modified by environmental and hormonal factors including stress and inflammation [14]. The migraine aura is due to the phenomenon of cortical spreading depression that can be triggered by various cortical lesions and is likely to be involved in the neurocysticercosis cases mimicking migraine with aura. Brain inflammation can modify cortical excitability through biochemical and glial changes in the neuronal environment resulting from breakdown of the blood–brain barrier [15]. IL-1β and Monocyte Chemoattractant Protein-1 (MCP-1) can powerfully modulate synaptic transmission by enhancing excitatory synaptic transmission and suppressing inhibitory synaptic transmission [15]. Calcified and granulomatous neurocysticercal lesions promote intermittent inflammation in the surrounding parenchyma, as demonstrated by MRI studies [16]. This phenomenon is probably due to intermittent antigen presentation to host immune cells during remodeling of chronic lesions, and has been correlated to epileptic seizures occurrence in patients with epilepsy and calcified neurocysticercosis brain lesions [17]. Our patient, however, had no migrainous auras and there was neither clinical nor EEG evidence of seizure activity. Although it was speculated on the basis of animal experiments [18] that subclinical cortical spreading depression might occur in migraine without aura, there is no evidence for this in human studies and such a mechanism is thus highly unlikely in our patient.

The migraine headache is thought to be due to activation of trigemino-vascular system, i.e. the major pain-signalling sytem of the brain consisting of trigeminal visceral nociceptive afferents innervating the meninges and their relay in the brain stem, the spinal trigeminal nucleus [19]. The trigemino-vascular pathway can be sensitized by inflammation [20] and migraine is known to be comorbid with a number of inflammatory disorders [21]. In multiple sclerosis that is a model of recurrent CNS inflammation, migraine is two to three times more frequent than in the general population [22,23].

Secondary headaches can mimic migraine as seen classically with post-ictal headache or headache attributed to arterioveinous malformation [7]. It remains to be determined if a focal inflammation like the one induced in neurocysticercosis is able to repeatedly trigger the trigemino-vascular system, and thus migraine headaches, in every individual or if this occurs only in individuals who have a genetic predisposition for migraine without aura [24]. Though our patient had no known family history of migraine, we cannot exclude such a genetic predisposition and thus the possibility that his neurocysticercosis was merely an aggravating factor of the migraine and not its cause.

## Conclusion

We report a case of neurocysticercosis presenting as episodic migraine. We discuss the possible relations between neurocysticercosis and migraine taking into account present knowledge on their pathomechanisms. We propose that in our patient the occipital neurocysticercosis lesions cause migraine without aura-like attacks via inflammation in the surrounding brain parenchyma leading to sensitization of the trigemino-vascular system and/or, less likely, subclinical cortical spreading depression waves. We cannot rule out, however, the possibility that our patient has a genetic predisposition for migraine without aura and that the fortuitous association of neurocysticercosis is simply an aggravating factor of his migraine. From a practical standpoint, this case report has potential clinical implications in the approach to patients presenting with recurrent headaches in the setting of high neurocysticercosis prevalence.

## Patient consent

Written informed consent was obtained from the patient for publication of this case report and any accompanying images. A copy of the written consent is available for review by the Editor-in-Chief of this journal.

## Competing interest

The authors declare that they have no competing of interest.

## Authors’ contributions

Acquisition of data: FY, CM. Drafting of the manuscript: FY, CM. Critical revision of the manuscript for important intellectual content: FY, DAG, NMM. All authors read and approved the final manuscript.

## Pre-publication history

The pre-publication history for this paper can be accessed here:

http://www.biomedcentral.com/1471-2377/14/138/prepub
